# Impact of forced diuresis on retropulsion of disintegrated ureteral calculi during semi-rigid ureteroscopy: a double-blind randomized-controlled study

**DOI:** 10.1007/s00240-022-01324-3

**Published:** 2022-04-22

**Authors:** Essam A. Shalaby, Khaled M. Abdelhalim, Mohamed Bakr, Ahmed A. El-Lilly, Mohamed A. Elkoushy

**Affiliations:** 1 Department of Urology, Suez Canal University, Ismailia, Egypt; 2 Department of Urology, Port-Said University, Port Said, Egypt; 3 Department of Anesthesia, Suez Canal University, Ismailia, Egypt

**Keywords:** Ureteroscopy, Ureteral stone, Diuretics, Stone retropulsion, Lithotripsy, Anti-retropulsion devices

## Abstract

The objective of this study is to assess the safety and efficacy of forced diuresis as an antiretropulsion strategy during the pneumatic disintegration of solitary lower ureteric stones with semi-rigid ureteroscopy (URS). A prospective randomized double-blind study was carried out from March 2019 to June 2021 for patients presented with unilateral solitary radiopaque lower ureteric stones ≤ 20 mm. Patients were randomized for URS into two groups, according to the use of forced diuresis using furosemide 1 mg/kg (GII) or not (GI). Perioperative parameters were compared between both groups, including retropulsion rate, stone-free rate (SFR), and need for auxiliary procedures and complications. A total of 148 patients were included; 72 (48.6%) in GI and 76 in the GII (51.4%), with respective stone size of 11.8 ± 2.6 vs.12.1 ± 2.4 mm. Both groups were comparable in demographic and baseline data, with a mean age of 47 ± 16 and 50 ± 14 years for GI and GII, respectively. GII had a significantly shorter disintegration time (10.5 ± 1.3 vs. 4.2 ± 2.1 min, *p* < 0.001), shorter operative time (33.1 ± 10.1 vs. 40.8 ± 9.1 min, *p* < 0.001), lower stone fragments migration rate during disintegration (6.5% vs. 18.1%, *p* = 0.04), lower retropulsion rate (1.3% vs. 11%, *p* = 0.02), higher SFR (96.1% vs. 86.1%, *p* = 0.04), and lower auxiliary procedures (3.9% vs. 13.8%, *p* = 0.03). Intraoperative and 6-h postoperative changes in heart rate and mean systolic blood pressure were comparable between both groups. Ephedrine injection (6–18 mg) was needed in significantly more GII patients (39.5% vs. 20.8%, *p* ≤ 0.01). It seems that forced diuresis during pneumatic lithotripsy of the lower ureteric stones is a safe and effective antiretropulsion technique. This would expand the alternative options to the antiretropulsion strategy, especially in centers where the laser and flexible ureteroscopes are not available.

## Introduction

Semi-rigid ureteroscopy (URS) is a safe and minimally invasive procedure for the management of distal and mid-ureteral stones with a success rate of 80–95% [1, 2]. The stone-free rate (SFR) is influenced by stone size, stone location, lithotripsy energy, the use of antiretropulsion devices (ARD), and surgeon experience [2]. The retropulsion of ureteral stone and migration of stone fragments into the kidney may necessitate re-treatment and serve as a source of persistent infection and stone recurrence [2, 3]. The retropulsion of the stone is further enhanced by the irrigation pressure, the stone location, the lithotripsy modality, and the ballistic effect of the probe as well as the degree of proximal ureter dilatation [3, 5].

Different antiretropulsion strategies have been used to prevent and minimize stone migration, including stone pinning, reverse Trendelenburg positioning of the patient, proximal placement of Lidocaine gel, and reverse thermos-sensitive gel [5]. However, retropulsion rates of 10–40% for distal and proximal ureteric stones have been reported, respectively [4, 5]. Although, the use of ARD such as Stone Cone and the NTrap, stone debris passing ARD, finally missed in the kidney and necessitating and the need for other procedures with subsequent increased cost [6, 7], especially when the flexible URS is not available [3, 4].

Intravenous administration of potent loop diuretics, with rapid onset of action was safe, efficient, and associated with a significantly higher SFR and decreased the average number of sessions per stone during extracorporeal shock wave lithotripsy (SWL) [8]. Antegrade saline irrigation through a nephrostomy tube and continuous saline irrigation through a ureteric catheter advanced above the stone level during pneumatic lithotripsy had achieved a higher SRF, less retropulsion, a clear lithotripsy, facilitates the spontaneous passage of the stone fragments and reduces the need for additional procedures [9, 10].

Therefore, the aim of the present study was to assess the safety and efficacy of forced diuresis with a loop diuretic as a tool of antiretropulsion during intracorporeal pneumatic lithotripsy, in semi-rigid URS for solitary lower ureteric stones in terms of retropulsion rates, lithotripsy and operative time, SFR, and the need for auxiliary procedures.

## Methods

After approval of the institutional review board no# 4394, Faculty of Medicine Suez Canal University Hospital, a prospective randomized-controlled study was conducted between March 2019 and June 2021 for patients presented with a unilateral solitary lower ureteral stone ≤ 20 mm in its largest diameter. Patients with a solitary functioning kidney, renal impairment, severe hydronephrosis, uncontrolled urinary tract infection (UTI), bilateral ureteral obstruction, concomitant renal stones, or congenital ipsilateral ureteral anomalies or stricture were excluded from the study. Patients in whom the stone was extracted directly without disintegration during URS or those experiencing a drop of systolic blood pressure of more than 25% of the baseline after anesthesia were also excluded [11].

Demographic and preoperative data were collected, including patients and stone characteristics. Patient parameters included gender, age, clinical presentation, body mass index (BMI), presence of medical comorbidity, current medical history, previous passage of stones, and previous ipsilateral procedures. Stone parameters assessed by non-contrast enhanced computed tomography (NECT) included size, location, laterality, multiplicity, density, and degree of the associated upper urinary tract dilatation. Full laboratory work-up was performed to assess comorbid conditions and fitness for anesthesia, including complete blood count, renal and liver profiles, bleeding profile, serum electrolytes, and a complete urinalysis and urine culture were essential before intervention.

NECT with transabdominal ultrasound (US) were performed for all included patients. Hydronephrosis was defined as fissuring of the normally echogenic central renal complex (mild), dilated pelvis beyond the sinus with uniform dilated calices with an average parenchymal thickness (moderate), and thin parenchymal thickness compared to the other side (severe) [12]. The lower ureteral calculi were defined as the stone located below the upper border of the sacroiliac joint [13]. The size of the stone was calculated by measuring the maximum longitudinal diameter of the stone in NECT. The stone-free status was defined as the complete removal of the stone or the presence of a non-obstructed residual fragment of ≤ 3 mm [14].

Patients who met the inclusion criteria were randomized into two-treatment groups depending on the use of forced diuresis (GII) or not (GI). The stratified block randomization was used, depending on stone size, to guarantee the fair and comparable distribution of different size stones on both groups. Randomization was performed by the anesthetist (A.A.E) in the operating room just before the procedure, while patients and surgeons were blinded in the randomization process. Patients were asked to refrain from eating solid foods for just 6 h before surgery, refraining from clear fluids was only 2 h prior to surgery to avoid the preoperative dehydration and hypotension.

### Surgical procedure

All procedures were performed under spinal anesthesia by two expert endourologists, after administration of preoperative prophylactic antibiotics. For GII patients, intraoperative furosemide was injected at a dose of 1 mg/kg 5 min after induction of spinal anesthesia with a maximum dose of 80 mg. Crystalloid co-loaded was done according to goal-directed therapy in both groups to maintain the intravascular fluid volume and mean arterial blood pressure above 80 mmHg [15]. The heart rate and blood pressure were measured at 0 min, which was just before spinal anesthesia and at 2, 5, 30 min and 1, 2, 4 and 6 h after spinal anesthesia. Atropine was given if heart rate decreases 20% of the baseline in a dose of 0.4 mg, which was increased by 0.1 mg IV boluses if heart rate did not reach the desired level. Ephedrine was given as 6 mg IV if systolic or diastolic blood pressure decreases by 20% or more from the baseline, and the dose was repeated every 2–3 min if desired response was not reached. Extra fluids were administered at a dose of 7 mL/kg in hypotensive crises [15]. In the lithotomy position, cystoscopy was performed with the introduction of a sensor guide wire, and retrograde ureteropyelography (RUP) was performed, guided by real-time fluoroscopy. An 8 Fr semi-rigid ureteroscope (Karl Storz Endoscopy, Tuttlingen, Germany) was introduced up to the level of the stone with intracorporeal pneumatic lithotripsy (Swiss Lithoclast-EMS Medical, Switzerland). At the end of the procedure, a double-J ureteral stent was inserted in all patients and was removed postoperatively after 2 weeks under sedation, in the absence of ureteral injuries, significant residual fragments and retropulsed stones to more proximal levels. The entire procedure was monitored by real-time fluoroscopy, and postoperative RUP was performed, when indicated, to asses for ureteral injury.

### Outcome measurement

Intraoperative and postoperative parameters were compared between both groups, including changes in heart rate and blood pressure, serum electrolytes, and operative time, length of hospital stay, and need for postoperative analgesia. Stone retropulsion was defined as; the migration of a significant residual fragment of ≥ 4 mm to the kidney during stone fragmentation, assessed with intraoperative fluoroscopy and postoperative NECT. Postoperative complications were recorded, suing the modified Clavien–Dindo Classification System [16]. Patients with residual stone fragments and/or stone retropulsion were monitored until the stone-free status, and need for auxiliary procedures.

### Data analysis

The data were analyzed using the Statistical Package for Social Sciences (SPSS) Version 23.0 (IBM Corporation, Armonk, NY, USA). At a 95% level of confidence and a 90% study power, sample size was calculated to detect an expected difference of 10% in the SFR between both groups. This yielded a total sample of 144 patients, including 72 patients per each arm, after considering a 10% dropout or missed follow-up patients. Categorical and continuous variables were presented as frequencies (percentages) and means (SD), respectively. Categorical variables were compared using Fisher’s exact test, while continuous variables were compared with the Student independent *t* test or Mann–Whitney *U* test, depending on the normality of data distribution. Mixed-design ANOVA were used to test the differences in the repeated measurements of the heart rate and blood pressure. A *p* value less than 0.05 was considered statistically significant.

## Results

**Fig. 1:**
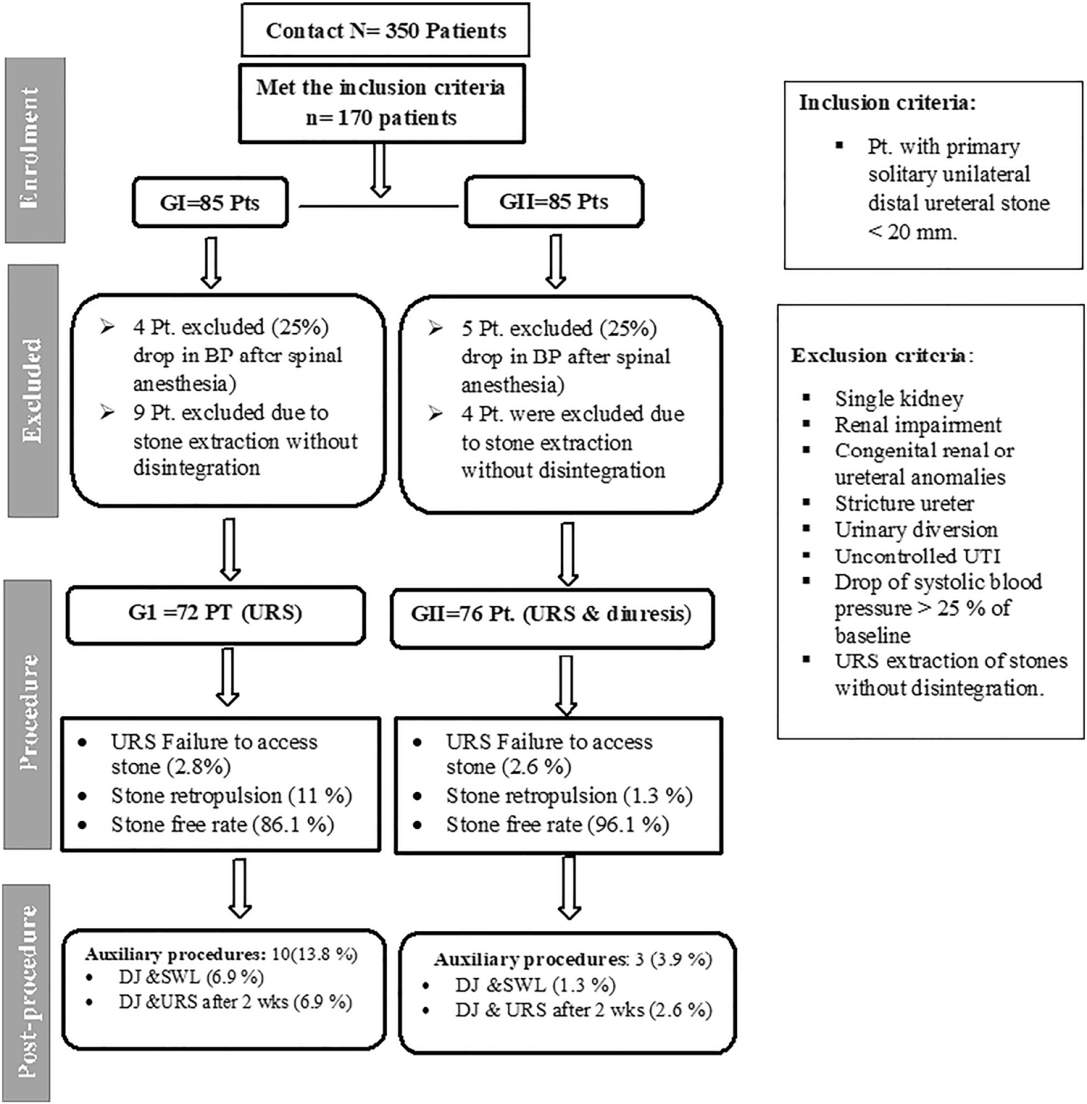
Flowchart of the study population

A hundred and seventy patients met the inclusion criteria, including 85 patients in each group. After the induction of spinal anesthesia, 22 patients were excluded; nine patients had a 25% drop in blood pressure from the baseline and 13 had stone extraction without disintegration. Therefore, 148 patients were included in the data analysis; 72 (48.6%) in the GI and 76 in the GII (51.4%) (Fig. 1).

**Fig. 2:**
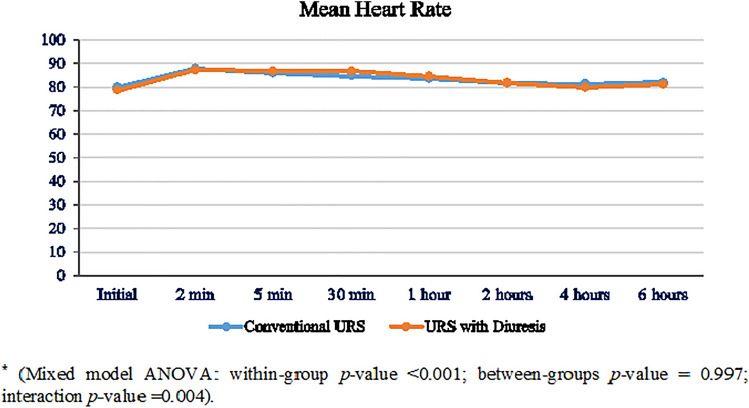
Change in the intraoperative and early postoperative mean heart rate in both groups*

**Fig. 3:**
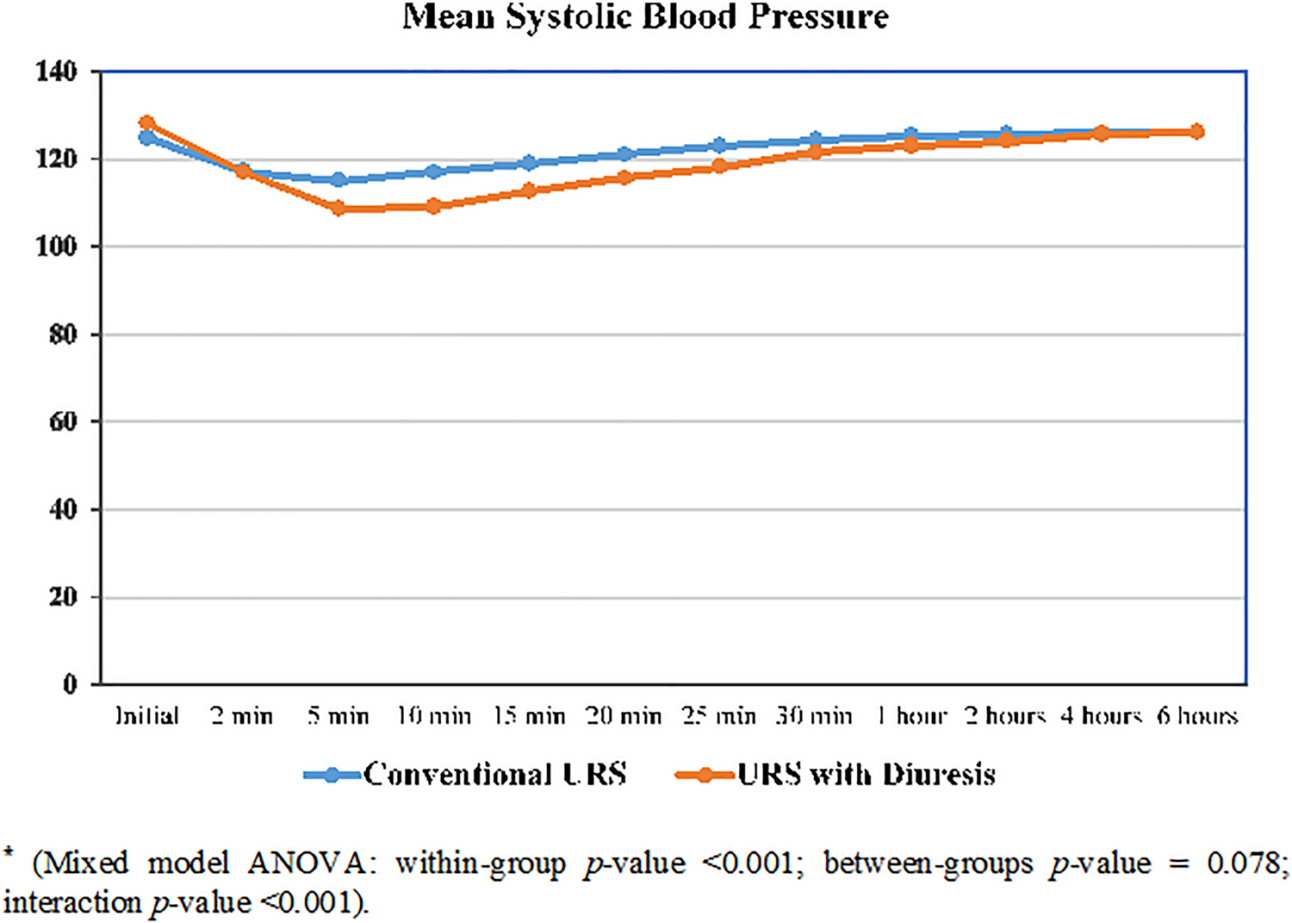
Change in the intraoperative and early postoperative mean systolic blood pressure in both groups*

**Fig. 4:**
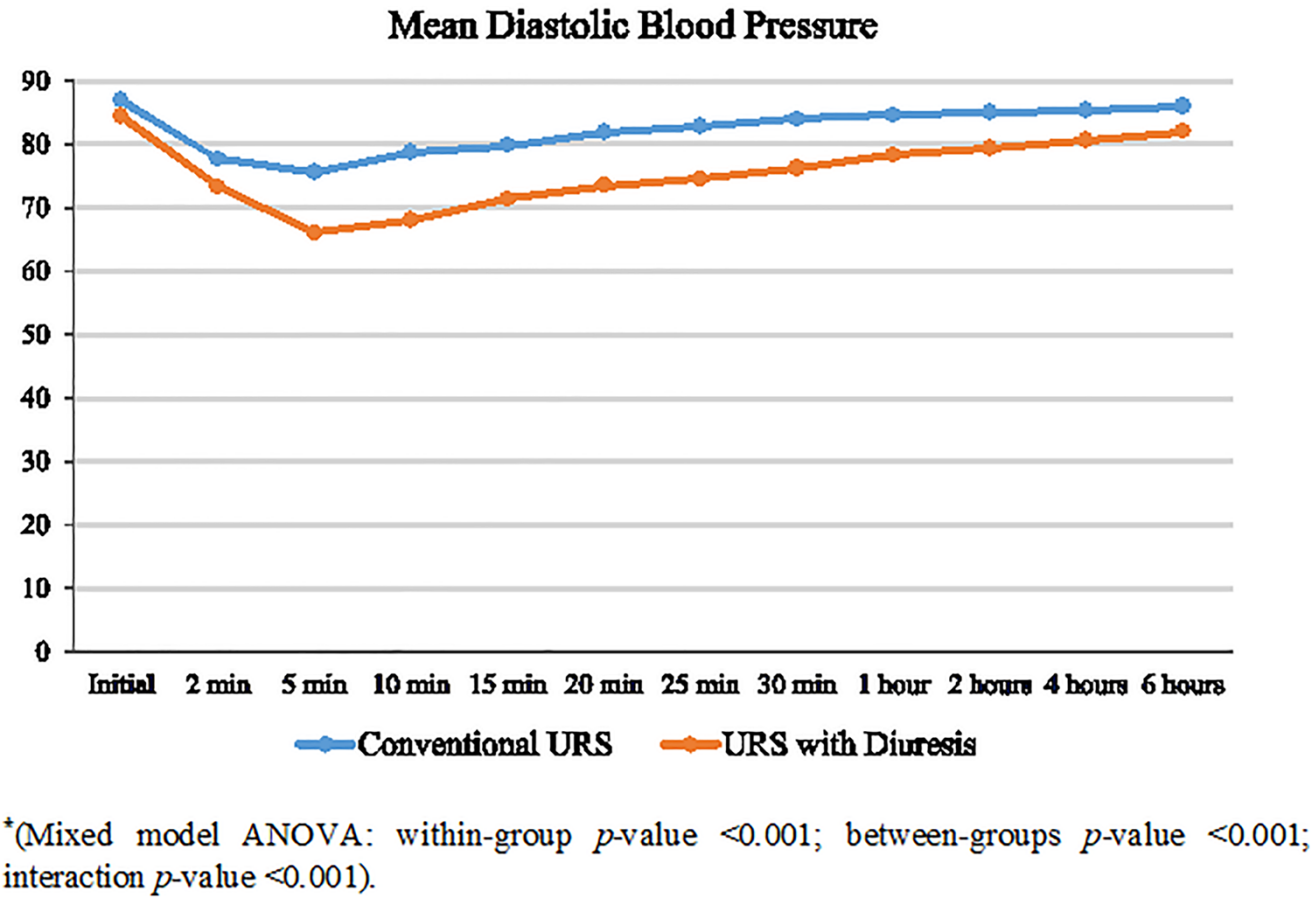
Change in the intraoperative and early postoperative mean diastolic blood pressure in both groups*

Both groups were comparable in demographic and baseline data, with a mean age of 47 ± 16 and 50 ± 14 years in GI and GII, respectively (Table 1). Patients in GII had a significantly shorter disintegration time (10.5 ± 1.3 vs. 14.2 ± 2.1 min, *p* < 0.001), and significantly lower operative time (33.1 ± 10.1 vs. 40.8 ± 9.1 min, *p* < 0.001). Proximal upward migration of stone fragments within the ureter during disintegration was significantly higher in GI patients (18.1% vs. 6.5%, *p* = 0.031). Migrated fragments were more significantly retrieved in the GII patients (80% vs. 38%, *p* = 0.29). Stone retropulsion of fragments ≥ 4 mm was significantly higher in the GI (11% vs. 1.3%, *p* = 0.02). Significantly more patients in GI required longer ureteral stent placement (23.6% vs. 10.5%, *p* = 0.03). Patients in GII had significantly SFR (96.1% vs. 86.1%), shorter length of hospital stay (0.8 ± 0.4 vs. 1.0 ± 1.4 days), and lower postoperative analgesia. Both groups were comparable in terms of low-grade postoperative complications (grade I–II) (Table 2). Intraoperative and 6-h postoperative changes in heart rate and mean systolic blood pressure were comparable between the two groups. However, GII patients experienced significant decrease in the mean diastolic blood pressure and needed significantly higher ephedrine injection (39.5% vs. 20.8%, *p* ≤ 0.01) (Figs. 2, 3, 4).

**Table 1:** Baseline data and stone characteristics

Demographic data	URS GI (*n* = 72)	URS + diuresis GII (*n* = 76)	*p* value
Age (years)	47 ± 16 (20–77)	50 ± 14 (20–75)	0.23
Female gender	41 (56.9%)	42 (55.3%)	0.84
BMI (kg/m^2^)			
Normal	28 (38.9%)	31 (40.8%)	0.10
Overweight	20 (27.8%)	29 (38.2%)	
Obese	24 (33.3%)	16 (21.1%)	
Left side	41 (56.9%)	51 (67.1%)	0.24
Stone burden			
Stone length (mm)	11.8 ± 2.6	12.1 ± 2.4	0.47
Stone volume (mm^2^)	143.6 ± 63.1	160.3 ± 67.9	0.12
Hydronephrosis			
Grade I	55 (76.4%)	60 (78.9%)	0.84
Grade II	17 (23.6%)	16 (21.1%)	
Preoperative serum sodium (mEq/L)	139.2 (± 1.7)	139.1 (± 1.8)	0.73
Preoperative serum potassium (mEq/L)	4.4 ± 0.55	4.3 ± 0.46	0.23

Data presented as mean ± SD (range) or frequency (%)

**Table 2:** Perioperative measures in both groups

Perioperative variables	GI *n* = 72	GII *n* = 76	*p* value
Volume of irrigation fluid (L)	5.5 ± 1.4	3.3 ± 1.6	< 0.001
Stone lithotripsy time (min)	14.2 ± 2.1	10.5 ± 1.3	< 0.001
Operative time (min)	40.8 ± 9.1	33.1 ± 10.1	< 0.001
Length of hospital stay (days)	1.0 ± 0.4	0.8 ± 0.4	0.003
Proximal fragment migration	13 (18.1)	5 (6.5)	0.04
Retropulsion (≥ 4 mm)	8 (11)	1 (1.3)	0.02
Intraoperative complications	7	5	0.76
Abortion of the procedure	2 (2.8)	2 (2.6)	0.77
Ureteral perforation	1 (1.4)	1 (1.3)	
Mucosal injury	4 (5.5)	2 (2.6)	
Postoperative analgesics			
≤ 1 amp/day	45 (62.5)	60(78.9)	0.03
≥ 2 amp/day	27 (37.5)	16 (21.0)	
Stone-free rate	62 (86.1)	73 (96.1)	0.04
Auxiliary procedures			
Total	10 (13.8)	3 (3.9)	0.03
SWL	5 (6.9)	1 (1.3)	
URS	5 (6.9)	2 (2.6)	
Number of ephedrine given (6:18 mg)	15 (20.8)	30 (39.5)	< 0.01
Postop serum sodium (mEq/L)	139.5 ± 1.6	137.4 ± 1.5	< 0.0001
Postop serum potassium (mEq/L)	4.3 ± 0.63	3.8 ± 0.4	< 0.0001
Postoperative complication			
UTI	3 (4.1)	1 (1.3)	0.48
Hematuria	2 (2.7)	2 (2.6)	

Data presented as mean ± SD (range) or frequency (%)

*UTI* urinary tract infection

The early postoperative decrease in serum sodium and potassium in the GII patients did not have clinical significance, where the differences were within the average. Significantly, more patients needed auxiliary procedures in GI (13.8% vs. 3.9%), including SWL (6.9% vs. 1.3% %, *p* = 0.03 (Table 2).

## Discussion

Semi-rigid URS represents an effective treatment option for ureteral stones with a success rate of 85–94%. However, stone location significantly influences the SFR during URS [1]. Backpressure effect of irrigation or pneumatic lithotripsy may affect unintentional proximal stone migration during URS [1, 5], with a retropulsion rate of 3–15% during the fragmentation of distal ureteral stones [17, 18]. These results in a lower SFR, a longer operating time and a higher incidence of residual stone fragments, resulting in UTI, recurrent stone growth, and increased the need for additional procedures such as flexible URS and SWL, with increasing morbidity and healthcare costs [3, 5, 19]. ARDs, such as stone baskets, suction devices, balloon catheters, guide-wire devices (Stone Cone and NTrap), and gel-based devices, have been used to overcome the retropulsion of stones during URS and improve the SFR [5]. However, the global use of ARDs does not exceed 14.5% [19]. In the developed countries, laser lithotripsy usually replace the use of ARDs, with a relatively lower stone retropulsion rate [19]. However, the cost of laser machine and its operating fibers limit its use in countries with modest resources in the developing countries [20]. Moreover, ARDs add extra costs for the operative procedure and have their own limitations [5, 21], including limitation of the endoscopic maneuver, accidental trapping of fragments, ureteral injury, and obscuring the clarity of vision, especially with the gel-based devices [21]. The use of pneumatic lithotripsy with limited access to flexible ureteroscopes stimulates the use of antiretropulsion maneuver [20].

Diuretic therapy was extensively used during SWL for ureteral and renal stones, with significantly higher stone fragmentation rate and SFR [8, 22, 23]. Therefore, forced diuresis was used the present study as a simple maneuver to reduce the rate of proximal stone migration through increasing the urine flow from the kidney. Ultimately, improving the visual clarity during active stone disintegration, increasing the washout of the smaller fragments and improving the SFRs. Furosemide is a potent loop diuretic, start action within 5 min of IV administration, with peak effect in 15–120 min and its duration of action may persist for up to 4–6 h [8]. Compared to GI patients, those undergoing forced diuresis during URS had significantly shorter disintegration and operating time, less intraoperative irrigation fluid, and significantly higher SFR. The diuretic force induced by furosemide seems to improve the clarity of vision, permit better disintegration, and increase the wash out of the disintegrated fragments. While an SFR of 83–95% and a retropulsion rate of 2–15.4% have been reported with stone Cone and NTrap devices [24, 25], the forced diuresis during URS has not been previously assessed.

In the current cohort, forced diuresis during URS improved SFR than the previously reported reverse Trendelenburg positioning, with a lower retropulsion rate (1.3% vs. 6.8%) [26]. Sun and Peng simultaneously used continuous saline irrigation during ureteroscopic lithotripsy by advancing the ureteric catheter above the stone level. The authors achieved a better SFR (100% vs. 92%) and a lower retropulsion rate (2% vs. 20%) than their control group, with a clear vision during lithotripsy and improved small fragments washout [10]. Jung et al. compared the antegrade saline irrigation using a nephrostomy tube verses retrograde irrigation during pneumatic lithotripsy. The antegrade group had a significantly higher SFR and shorter operation time [9]. It seems that the antegrade flow of fluids during disintegration prevents stone retropulsion facilitates the spontaneous passage of stone fragments and reduces the need for additional procedures. Using the stone cone and the N-Trap was associated with a retropulsion rate of 2.1% and 2.9%, with an SFR of 97.1% and 95.7%, respectively [24]. Similarly, using the Dormia basket and the stone cone achieved a retropulsion rate of 2.3% and 8.3%, with an SFR of 91.7% and 97.7%, respectively [20]. Although improving the SFR during URS, ARDs brings extra costs, which may not be effective for all ureteral stones, especially if the retropulsion rate is less than 6.3% [27].

In the present study, patients undergoing forced diuresis have significantly lower proximal stone migration during disintegration, with more successful removal of the migrated fragments. Intraoperative adverse events were comparable between patients in GI and GII, including ureteral mucosal injury (5.5% vs. 2.6%) and ureteral perforation (1.4% vs. 1.3%), respectively. Others reported a mucosal injury rate of 10.5–13.9%, with a 1.4% ureteral perforation rate [20, 24].

Notably, furosemide may intensify the hypotensive effect of the spinal anesthesia, which necessitate perioperative prevention and treatment. Despite the significantly decreased mean diastolic blood pressure in the GII patients, it did not have equivalent clinical significance. The use of ephedrine as a fast-acting vasopressor with co-loaded crystalloids and appropriate fluid replacement are crucial in the management of these hypotensive crises [12, 15, 28]. The significantly shorter hospital stay and a decrease in analgesic requirements in GII patients may be due to the significantly shorter operating time they had.

The main limitations of our study may reside in the pneumatic intracorporeal lithotripsy, which may not be practical with laser lithotripsy. However, this maneuver may have the same, or even more, benefits if used with laser lithotripsy, due to the smaller fragments resulting from disintegration. Of note, laser and flexible ureteroscopes may not always available, especially in the developing countries. In addition, this is the first study to provide the advantages of forced diuresis as a new and safe retropulsion maneuver during the pneumatic ureteral stone disintegration.

## Conclusion

Forced diuresis during pneumatic lithotripsy of the lower ureteric stones during semi-rigid ureteroscopes is a safe and effective antiretropulsion technique. This would expand the alternative options to the antiretropulsion strategy, especially in centers where the laser and flexible ureteroscopes are not available.

## Abbreviations

ARD: Anti-retropulsion devices
BMI: Body mass index
HN: Hydronephrosis
IV: Intravenous
PNL: Percutaneous nephrolithotomy
RUP: Retrograde ureteropyelography
SFR: Stone-free rate
SWL: Shock wave lithotripsy
URS: Ureteroscopy
US: Ultrasonography
